# Supplementary material for: Patterns of Intron Gain and Loss in Fungi
Source: PLoS Biol. 2004 Nov 30;2(12):e422. doi: 10.1371/journal.pbio.0020422 (PMC532390; doi:10.1371/journal.pbio.0020422)
Supplement: Table S1 — Also available at http://genes.mit.edu/NielsenEtAl/. (4.3 MB ZIP). [file pbio.0020422.st001.zip › NielsenEtAl/html/1238.html]

AN2925.1.NCU08373.1.MG00529.1.FG05596.1


```
 CLUSTAL W (1.82) Multiple Sequence Alignments - Introns Inserted


Sequence 1: NCU08373.1	1381 aa
Sequence 2: MG00529.1	1375 aa
Sequence 3: FG05596.1	1139 aa
Sequence 4: AN2925.1	1513 aa
Alignment Length: 1545 aa
Number Identitical Residues: 484 aa
Alignment Score (without introns) 25626


MG00529.1 	-----------MDPAQTRKRRRRRRQDKLPISARLALDDHVRRDVGILSEDLFADLF--P
NCU08373.1	------MTAPNSTPASSRKRVRRRRQDKPALSAKLVLDAHVKGDVGVIATDLFADLF--P
FG05596.1 	------------------------------------------------------------
AN2925.1  	MDLERHEFSSDQSIRRRRNFARKRLRNKTALSARLSLDHRLRGSVGVVSDDLANDLFQPP
          	 . .    :..       .       .. . ::    .     . .  : .   .  ...

MG00529.1 	HLSP~D~EVHED1DVYYVAISPWIPG-ATPESIDWTIVAVTR-------STALSHSTVQF
NCU08373.1	HLWA~N1-GDSQ~DTVHVAIAPWAPS-QTPEANNWSIVPVIR-------SSTVAPSTVQF
FG05596.1 	----~-~-----~-----------------------------------------------
AN2925.1  	DPTD1D~FQDDS~GIRYIAISPYLPGPSSVEALTWTIVPVRPQSAGRSNESPLPKSTIIF
          	     .    .. .    : :.  ... : .:   :  .   .::. :..:. . ::   

MG00529.1 	SPSASALALQTFATSLQRVAPSKLSSHSRSGIDILVLDALALSLDTVFVSLDGELAKRLE
NCU08373.1	SPSS--LALQSFATHLQQAAPSKLSGHNKGGIEILILDVSPIELDTVFVNLDGELAKRLE
FG05596.1 	------------------------------------------------------------
AN2925.1  	PDSAD--SLQPFVRALSKLDPTRQSPHTLRLIEIRVMDVVPLPLDTIYVTVERDLLRNHD
          	. ::.  : ..     .   .:  :  .    .    .  .   .:   . . .     .

MG00529.1 	HGEGVFHR----------------EHPIG-KGSATP-PSPEQKLAAAVRAALGTLRVVHS
NCU08373.1	DGEGTFFR----------------EHPVNGKGKARADATPEEHLTAALRTALGTLKIVHS
FG05596.1 	------------------------------------------------------------
AN2925.1  	DVQSRFGGGFTSNMNGPNGLWAKGKGAESKKYSKKAAAEVEGRLTAAVREALGTQRVVHT
          	  ..    . ::. .....  :... . . . .  . .  .   :::   : .:     :

MG00529.1 	GDLFPLPLPPHPITHVPPNPGKVTLCEPVAQGILSPNTKIIVSSGRSPLKGNRDRGSRQS
NCU08373.1	GDFFPLPLPPHPVTHQPPNPGRITLCEPVAQGILAPTTKIIVSRVRTSKTKRSSASGQQQ
FG05596.1 	------------------------------------------MRGRVHTKQIRRVVSVTA
AN2925.1  	GDSLPLPLPPHPITYAPAPPALISFCEPVSQGLLLPTTKIVLIQARPHGNRAQRSLRPAS
          	..  . . .. . :  .. ..  : ... :..   ..:.      *   .          

MG00529.1 	SRMLDGVAEADEDTANDAFFSAAEERYKTEASETADESAATSASETELSSGDIDDLSDDS
NCU08373.1	GRKLNGVAEDDED----AYFSAAEEDRDSKTNAQTAETDADDTEFEPQAGEDEDNLSDDS
FG05596.1 	SQQLNGVPEDEDDTSTDQFYSAAEDR--DKSGMIEDMESATETEPDLSDFENEDELSDDS
AN2925.1  	GFLKQVAEDEADDTSNEQFYSAAEDKPVDDSTEMESTSNAEESETEGS-GGNTSDTSDDS
          	.   : . :  :*::.. ::****:    .:        * .:.       : .: ****

MG00529.1 	LDDMISLQTPMLPSN-ASGISTAQAGTPMTIG--RGRKTNGMATPGSVFSSFTATTARPD
NCU08373.1	LDEIISLQVPTLPTT-VTGQSTIGTGTP-TML--RGRKTNG---PGSVISSYTATTARPD
FG05596.1 	MDDMISLQAPTLPTTNASGVSTLQPGTPMTVGTLRGRKTNGITTPGSVFSNYTATTARPD
AN2925.1  	LDDMISLSAPELPQPATGVMSGMTSATP------RARRMDGIHTPGSMVSNLTSSTLRPG
          	:*::***..* **   .   *   ..**      *.*: :*  :***:.*. *::* **.

MG00529.1 	RPRG-RLFKAQGLVKPIPPELLHPKPSAEDDDEAHIYVDVSHLSRIGCFSGDWVRVEASA
NCU08373.1	RPRG-RLFKAHGLMKPIPPDLLHPKPTPEDDEEACIYVDMRDLTRVGCFSGDWIRVEAAS
FG05596.1 	RPRG-RLFKAQGLMEQIPADILHPKPSIEDDEEARIYVDVTSLSRIGCFSGDWVRIEAAD
AN2925.1  	RQGGGKVFKAEGLLRRVPNELLYPKPRDDDDVEAVVFVDISTLAKIGCFSGDWVRIEASE
          	*  *.::***.**:. :* ::*:***  :** ** ::**:  *:::*******:*:**: 

MG00529.1 	APPANGFGPFALGSFGEPDAQEPVWRPARVFGLPEGYSNRPVSRIPSSKHRGRSLSFFES
NCU08373.1	EPPSNGLGAFGLGSFVEQEADEINWRPARVFGLPEGYSSRSATRSHHSRHDSRNSSFFEA
FG05596.1 	EPPANGFGAFGLGSFGQAEPEPAKWRPVRVYGLPEGYSQRPMTRIPSAKHGERRLSFFES
AN2925.1  	EPQANIFSSINLGSFNEQYG-EGDWRAVKIYGLPGLPSAKPRYSIKQS--GDRRLSFSQR
          	 * :* :..: **** :       **..:::***   * :.      :    *  ** : 

MG00529.1 	QVQKSSGPLAYASPILLANLDLPSHLRLLPLKRTIP--HHGNGPQSQATAIVQPPYARGV
NCU08373.1	QSQKSN-PPAYTSPILLANLDNPPYLRLSPIKRPSSSIIKGVAAQSKTSSTLKPPFAREV
FG05596.1 	QIQRPASPTAYASPILLANLDDAPYLRLAPLKKSSY---QGKGTIPRFTSAARPPYARDI
AN2925.1  	PGVRMT-PSVFVPPLLLNNMDNPRYLRISPMSLGGIGVPKSGVLHQMKTAARSPPVAKEV
          	   :   * .:..*:** *:* . :**: *:.    .  :.       ::   ** *: :

MG00529.1 	VVQQVRTPLTSSKDVEVAVIAGLKWHFEKKLRVIKAGDLIAVPIDTQLGRTMRETSN-GE
NCU08373.1	ILQHVRTPTAVERDVQSAVMAGLKHYFERRLRVLRTGDLIAVPIDTQLGKALQESTIPGE
FG05596.1 	TIHHIRTPISAERAFQTAVLGGLKRYFAQKIRLVRSGDLIAVPVDAELGRTLQEMPG-AG
AN2925.1  	TLLKVSTPVSMDRALQPALFSALKQYFESKRRLLKSGDLLGISIDETLGRAVFAGTG--A
          	 : :: ** : .: .: *::..** :*  : *::::***:.:.:*  **:::   .    

MG00529.1 	GSAIDNILSLAESSNSGTAASRRGLKYDEVAWFKVSHVQTQKQD-----AADQDEDLWGS
NCU08373.1	DSAIDEVLGLIAATRPG-----QSLHYDDVAWFRVGHVQAMKQDTTEAVDGEEAEDLWGG
FG05596.1 	GSEVDDVMALTVGGQETG---KQPSKFDSVAWFKVGHIQIHKSDED---EDGSAEAFWGS
AN2925.1  	DGQDDDITTKLGPGLDTN---RAGPKKIGVAWFRVGQVIPSLPED----QDDFGEFDWGG
          	..  *::                  :   ****:*.::     :          *  **.

MG00529.1 	AACVETTMAHFESSGMVNGRVPGVKDSNWPYYLGLVTPPNPGSD---SLPVAICEPAPKH
NCU08373.1	IACVDISLTHLEQSGSVTGRIPGTISNSWQYYLGIRKLPKHQQNPGLPAQLQTLEPEKQY
FG05596.1 	VACIDSSSVAMHGSGFETSRIPGIKQSTWQYYLGLKKTPKKPSE---TTPAPIQEPELPY
AN2925.1  	VAVIDSATTRMVQAGSDVSRIPNTLNNGWEYWLGTKSIPKPVVDT-QTIHGVVTEVPQPF
          	 * :: : . :  :*   .*:*.  .. * *:**  . *:   :.  .      *    .

MG00529.1 	ISALRRRMRSLLAAATSKRAIHLKAPAVAILLVSTQRNIGKATLATDTCRDIGLHTFTIN
NCU08373.1	VSSLRRKLRELMAAATSKPALHLNLPPLAILLVSTQRHIGKAATAMQACSDIGLHTFAID
FG05596.1 	VSPLRRQLRELIAAATSARAIHLKMPPVAVLLTSTHRNIGKSILATGACSDMGLHTYTID
AN2925.1  	VSPIQQRVRDLMAVATSSRAIQLGMKPVVILLRSQQRHIGKASLATRACAEIGLHTFPID
          	:*.:::::*.*:*.***  *::*   .:.:** * :*:***:  *  :* ::****:.*:

MG00529.1 	AYDILNEGGGGG-SDVKTDGTLKARAERALSCGPEFCALLIRHIEALTADRMATSLRSIL
NCU08373.1	AYDIVNDGGGGGGSDVKTAGFLTSRAERAMSCGPESCVLLVRHIEALTADRMVSSIKEIL
FG05596.1 	AYDILSEGSGSG-SDVKTEGFLRTRAERAMSCGSDCCALLVQHVEALTADRIESTIKEIL
AN2925.1  	AYDILTEGGANG-GDVKTEAYLKARAERAFHCGADCTALLIKHIEVLTADRIVSAMTEIL
          	****:.:*...* .**** . * :*****: **.:  .**::*:*.*****: ::: .**

MG00529.1 	SDARVLIATTTEPDKIPDGVRGLFTHELEMTAPDEGEREGILRSIVDQRGINLDPGVDLG
NCU08373.1	ADARVLIATTTEVEKVPDGIRALFTHELEMSAPDEQEREGILSSILADRGIGLDHGVDLS
FG05596.1 	EDTRVMIATTNEVDKVPDGVRALFTHELEMTAPDEAERESILKSIISDRGVSLEPSIDLN
AN2925.1  	ADARVIIATTTDVEQIPEAIRSMFTHEFELTAPEEKEREGILRNAVSERSIKLAPDVDLG
          	 *:**:****.: :::*:.:*.:****:*::**:* ***.** . : :*.: *  .:**.

MG00529.1 	GVALKTAALVAGDLVDVIDRAIVAQRSRLESLAEKASQSRQGNQPAVTLRDVQVAGGTAV
NCU08373.1	GIALKTAALVAGDLVDVVDRALVAQRSRLEKLTAKATGS-------ITFRDVQLAGGPAA
FG05596.1 	SIALKTAALVAGDLVDVVERASIAQQSRLEQLSAKNTHEN----TIITVRDVQVAGGPLA
AN2925.1  	TVALKTAALVAGDLVDVVERASVARSTRLEKLAESASKQSP--TSKITVRDVLLAGGEAV
          	 :***************::** :*: :***.*: . : .     . :*.*** :***  .

MG00529.1 	RGLTKGDLDTAVEAARKNFADAIGAPKIPSVTWDDVGGLGNVKDAVMETIQLPLERPELF
NCU08373.1	SGLTKQDFELAVDAARKNFADSIGAPKIPNVTWDDVGGLGNVKDAITETIQLPLERPELF
FG05596.1 	RCLTKSDFEIAVEAARKNFSDSIGAPKIPNVTWDDVGGLNNVKEAVTETIQLPLERPELF
AN2925.1  	RGVTKADFDAAVDAARKNFADSIGAPKIPNVSWEDVGGLTNVKDALVETIQLPLERPELF
          	  :** *:: **:******:*:*******.*:*:***** ***:*: *************

MG00529.1 	AKGMKKRSGILFYGPPGTGKTLLAKAIATEYSLNFFSVKGPELLNMYIGESEANVRRVFQ
NCU08373.1	AKGMKKRSGILFYGPPGTGKTLLAKAIATEYSLNFFSVKGPELLNMYIGESEANVRRVFQ
FG05596.1 	AKGMKKRSGILFYGPPGTGKTLLAKAIATEYSLNFFSVKGPELLNMYIGESEANVRRVFQ
AN2925.1  	AKGMKKRSGILFYGPPGTGKTLLAKAIATEFSLNFFSVKGPELLNMYIGESEANVRRVFQ
          	******************************:*****************************

MG00529.1 	RARDARPCVVFFDELDSVAPKRGNQGDSGGVMDRIVSQLLAELDGMSSGDDSGGGVFVIG
NCU08373.1	RARDARPCVVFFDELDSVAPKRGNQGDSGGVMDRIVSQLLAELDGMSGGEGGGGGVFVIG
FG05596.1 	RARDARPCVVFFDELDSVAPKRGNQGDSGGVMDRIVSQLLAELDGMSGGDDGGGGVFVVG
AN2925.1  	RARDARPCVVFFDELDSVAPKRGNQGDSGGVMDRIVSQLLAELDGMNGGEENSGGVFVIG
          	**********************************************..*: ..*****:*

MG00529.1 	ATNRPDLLDQALLRPGRFDKLLYLGVSDTHEKQLTIMEALTRK2FTLHPSVSLRAVAEKL
NCU08373.1	ATNRPDLLDPALLRPGRFDKMLYLGVSDTHDKQVTIMEALTRK2FTLHPTVSLRSVAERL
FG05596.1 	ATNRPDLLDPALLRPGRFDKMLYLGVSDTNDKQQTILEALTRK2FTLHPSVSLASVAEKL
AN2925.1  	ATNRPDLLDTALLRPGRFDKMLYLGVSDTHEKQATILEALTRK~FALAPDLSLARVSERL
          	********* **********:********::** **:****** *:* * :**  *:*:*

MG00529.1 	PFTYTGADFYALCSDAMLKAVTRQASHVDAKIKAMSAERQQE---ITTAYFFDHFATPED
NCU08373.1	PFTYTGADFYALCSDAMLKAVTRQATLVDTKIRELNAAAGPEGKQISTAYFFDHYATKED
FG05596.1 	PFTYTGADFYALCSDAMLKAVTRQATAVDNKIRAINADPETHHP-ISTAYYFDHYATPED
AN2925.1  	PLTYTGADLYALCSDAMLKAITRKATAVDEKIKQLPGG------PVSTAYFFDHLSTPDD
          	*:******:***********:**:*: ** **: : .        ::***:*** :* :*

MG00529.1 	IDVMVTEEDLLAAHEEMIPSVS~AGELAHYERVRATFEGGR--DKQQQQQQQRKD-----
NCU08373.1	ISVMVTEQDFLDAHRELVPSVS~AGELEHYEQVRAMFEGAK--DKDKKKEGAGGDGNGVD
FG05596.1 	IAVMVQEQDFLAANEELIPSVS~AGELAHYEHVRATFEGVRGTEKDNKPAPVQRAVSGAS
AN2925.1  	VAVMVTEEDFVAAQSELVPSVS2AKELEHFERIRQMFESTDKDKQKQDANPAPTPNTIGD
          	: *** *:*:: *: *::**** * ** *:*::*  **.    .:.:.        .  .

MG00529.1 	-LGARTV----------------SGASSVKGKGKGKGKAIAS-----------SSGGEEE
NCU08373.1	GLESGNR----------------NGMLAITMGDDGAGAESTK-----------KDGKGKG
FG05596.1 	TSSKGKG----------------KAVASGPGKGKGKGIALTS-----------DDEIDEE
AN2925.1  	AIEALKLGHDINGYIGDPVTNGGDQPLSPTGTGKGKGKQPAYGSMRSVSGQSVSSSRGKG
          	     . . . ..  ... :....   :    ..* *   : .:  : :..: ..   : 

MG00529.1 	DRQDGVNGKANKGKGKAVASGYG-SGGD-DEGLYD---------------------~---
NCU08373.1	KAADNEVNGAGEGKGKEGVSPFQESGGDEDEGLYD---------------------~---
FG05596.1 	DEVDDMNGQS-KGKGK-APMGFQDGTASDDEGLY----------------------~---
AN2925.1  	KAATSGGKSSLKSKSVSAANGNADSDSSLDGPLMGSGARGGVNSDGDDDDDDNDYI0LYN
          	.   .    : :.*.        .. .. *  * .:.: .. .:..........     .

MG00529.1 	--------------------------------~-------------------
NCU08373.1	--------------------------------~-------------------
FG05596.1 	--------------------------------~-------------------
AN2925.1  	LMLTYSRVIYVLASASIPTRLASVHFNWYIAR2HKLTFSASPFRAIGNPENT
          	   : :      :::: .:  ::   .   :   . : :::.  : .....:
```
